# Design and simulation of key components for mechanical transplanting of large pineapple seedlings

**DOI:** 10.3389/fpls.2025.1721367

**Published:** 2025-11-26

**Authors:** Junwang Guo, Beier Zhou, XiaoJie Liu, Zhenhui Hui, Xiaying Hao, Zhaoxin Zhang, Zhijie Yang, Haitian Sun, Zhong Xue

**Affiliations:** 1 College of Engineering and Technology, Tianjin Agricultural University, Tianjin, China; 2 South Subtropical Crops Research Institute, Chinese Academy of Tropical Agricultural Sciences/Key Laboratory of Tropical Fruit Biology, Ministry of Agriculture and Rural Affairs, Zhanjiang, China; 3 College of Engineering and Technology, Shanxi Agricultural University, Jinzhong, China

**Keywords:** pineapple, transplanting, mechanization, standardized treatment of seedlings, duckbill-type planting device, solid works, finite element analysis

## Abstract

Pineapple is an important characteristic crop in tropical and subtropical regions of China. In production, the proportion of labor costs is high and the level of mechanization is low, which hinders the full play of the industrial potential. Mechanized production of pineapple is the fundamental path to improve economic benefits and promote high-quality development of the industry. Among them, transplanting is a key technical link in the mechanized production of pineapple. Therefore, in order to improve the mechanization level of pineapple seedling transplanting in China, this paper reviews the existing key transplanting technologies and pineapple planting technologies and equipment at home and abroad, studies and analyzes the planting mode and physical characteristics of pineapple seedlings, standardizes the treatment of pineapple seedlings, and designs a duckbill-type pineapple seedling transplanter that can perform membrane transplanting operations. This study expounds the overall structure and working principle of the duckbill-type transplanter, optimizes the design of the transmission system and planting components of the transplanter, uses SolidWorks to conduct 3D modeling and interference analysis of the whole machine, and combines the actual force conditions to conduct finite element analysis of the core components. The analysis results show that there is no interference in the 3D assembly model, which meets the design requirements; the maximum strain, maximum stress and maximum displacement of the frame, driving shaft and transplanting arm all meet the design requirements, and the maximum stress is 2.816e+07Pa, 5.387e+08Pa and 1.448e+08Pa respectively, which does not exceed the yield strength of 710MPa of the 40CrNiMoA material after normalizing, verifying the correctness of the structural design and the safety of the mechanism strength and deformation. The results of the field experiments show that the pineapple seedling transplanting machine has a good transplanting effect, significantly improves transplanting efficiency, effectively reduces labor intensity, and lowers labor costs.

## 1 Introduction

Pineapple (Ananas comosus (L.) Merr.) is the third largest tropical fruit in the world and the fourth largest in China (Chen et al., 2021), with over 70 varieties. It is known along with bananas, papayas, and lychees as one of the four famous fruits in southern China. Due to its excellent fruit quality and scientific nutritional composition, the demand for pineapples continues to rise, promoting an increasing planting area of pineapples. The pineapple-growing areas in China include Guangdong, Guangxi, Hainan, Yunnan, Fujian, etc. Among them, Guangdong and Hainan are the main production areas (Liao et al., 2014). By the end of 2020, the total area of pineapple production in China was approximately 66,000 hectares, with a total output of about 3 million tons (Office of South Subtropical Crops Development, Ministry of Agriculture, 2020).

At present, the mechanization level of pineapple production in major production areas in China is relatively low, with a high reliance on manual operations in the transplanting process. Labor costs account for more than half of the total planting cost per acre. Moreover, the shortage of a large number of young laborers in rural areas has led to a continuous increase in labor costs (Qiu et al., 2025). This has become a key bottleneck restricting the large-scale and intensive development of the pineapple industry (Deng et al., 2019; He et al., 2024). The domestic pineapple market presents a situation where supply and demand are in conflict, with frequent price fluctuations. Under this situation, promoting the application of mechanized pineapple transplanting technology is of great significance. It can effectively alleviate the problem of labor shortage, reduce labor intensity, lower planting costs, achieve precise and standardized planting operations, increase economic benefits, stabilize market prices, and provide convenience for farmers’ production (Xue et al., 2021).

Although some pineapple transplanting equipment has been developed in China at present, the machines have poor adaptability to the environment and a low level of automation. The transplanting mechanism, as the core component of the transplanting equipment, needs to complete the operations such as extracting the seedlings from the seedling tray, transporting them, and planting them in the designated holes successively. The diversity of its functions and the stability of its performance directly determine the market competitiveness of the transplanting equipment (Yu et al., 2022). Currently, the transplanting technologies at home and abroad mainly include three categories: top-out type, clamping type, and direct-drop type. Their implementation forms cover mechanical, pneumatic, and mechatronic integration technologies (Wen et al., 2021). Among them, the mechanical transplanting mechanism includes planetary gear systems, multi-link mechanisms, and cam-rod mechanisms, etc (Lyu et al., 2017). However, in terms of its innovative design, most of China relies on experience accumulation, subjective intuition, or direct imitation of foreign transplanting mechanisms, which greatly restricts the research and development process of China’s independently intellectual property transplanting equipment. To meet the differentiated needs of seedling cultivation and transplantation under different crops and planting modes, developing high-performance and diversified transplanting mechanisms that match the physical characteristics and agronomic standards of the transplanted seedlings has always been a key direction in the research of transplanting planting machinery.

Therefore, based on the concept of integrating agricultural machinery and agronomy, this paper developed a duckbill-type pineapple transplanting equipment on the plastic film. This was aimed at breaking through the key technical bottlenecks of pineapple planting mechanization and achieving the integration of the process and improvement of efficiency for the bag-upright transplanting operation (Li, 2022). By deconstructing the “hole-making-planting-covering soil” three-station separation mode in the traditional transplanting process, a fully integrated system capable of planting on plastic films was pioneered. This reduced the step of opening the ground film hole (Han et al., 2015; Shao et al., 2019), and using SolidWorks to conduct finite element analysis on the frame, drive shaft, and transplanting arm, this structural design concept and method provided innovative references for the traditional design of pineapple transplanting machines and other agricultural machinery.

## 2 Pineapple agronomic cultivation techniques and design requirements

### 2.1 Cropping pattern

The combination of agricultural machinery and agronomy forms the foundation of mechanization. Only when both are developed in a coordinated manner can the functions of agricultural machinery and planting techniques be fully exerted (Jia, 2017). Currently, there are a wide variety of pineapple varieties in China, and the planting patterns in each pineapple-producing area are diverse. There is a lack of unified standards, which makes the mechanized field management and harvesting of pineapples quite challenging. Especially, the planting row spacing is too narrow, preventing machinery from entering the field for operations during the field management and harvesting processes of pineapples. To achieve mechanized pineapple production, based on the principle of combining machinery and agronomy, considering comprehensive economic benefits, and coordinating the research on mechanized technical ideas for pineapple planting, management, and harvesting, new suitable-machinable pineapple cultivation techniques should be developed (Cui et al., 2022).

In terms of pineapple agronomic cultivation techniques, pineapple should be planted in a reasonable density. However, due to the different planting requirements in each planting area, there are various pineapple planting patterns (Guo and Zhang, 2018), and there is no unified planting standard. Kossi et al. (2025) conducted a study to investigate the influence of ridge height and planting density on the growth and yield of pineapples under waterlogged soil conditions. This study found that in the Bafar region of Cameroon, with a 30 cm ridge height and a planting density of 57,000 plants per hectare, the highest fruit yield was achieved, at 87 tons per hectare. The research content showed that a 30 cm ridge height significantly enhanced root development, leaf area, and fruit yield. Compared with a 15 cm ridge height, the canopy yield increased by 149%. A 45 cm ridge height would form a capillary barrier about 15–20 cm below the ridge top, which would affect the water movement in the soil during wet and dry periods. In addition, increasing the planting density improved resource utilization efficiency without affecting growth parameters.

Dong et al. (2025) conducted a field mechanized transplanting experiment and identified the narrow-spaced and wide-spaced dense planting pattern with the greatest yield advantage in Northeast China (with row spacing and plant spacing of 36 + 14 cm×16 cm per row). Based on the effects of different planting patterns on the physiological and morphological characteristics of individual rice plants and the rice population, they revealed the significant influence of plant row spacing configuration on rice yield, photosynthesis, and material transportation properties. The mechanized transplanting of narrow-spaced and wide-spaced configurations not only increased yield but also effectively optimized the spatial structure of the population, improved resource utilization efficiency, enhanced production adaptability, and expanded the promotion potential.

Djido et al. (2021) conducted experiments and concluded that agronomic measures have a significant impact on fruit quality in pineapple cultivation. Based on the experimental results regarding the planting density (54,400, 66,600, and 74,000 plants per hectare) and the K_2_O:N ratio on the quality, shelf life, and yield of pineapple fruits, it is recommended that pineapple farmers in Benin adopt a planting density of 66,600 plants per hectare, combined with a potassium fertilizer application plan based on a K_2_O:N ratio of 1, to meet the expectations of producers and consumers for fruit yield and fruit quality.

Liu et al. (2024) conducted a study and summarized the “12(2)3” planting pattern for high-quality and efficient production of pineapples. This pattern suggests reducing the planting density of pineapples, reducing the planting density of the Bali variety from the traditional 60,000-67,500 plants per hectare to 45,000 plants per hectare, in order to improve the ventilation and light transmission conditions in the field, thereby effectively promoting the growth of pineapple plants and fruits, accelerating the accumulation of photosynthetic products, and ultimately achieving an increase in yield and quality. However, this study did not clearly provide specific parameters and requirements for plant spacing and row spacing during pineapple cultivation.

Sun (2020) reviewed the introduction performance and cultivation key points of Taiwan No. 16 pineapple in Guangdong Zhanjiang. It recommended the use of a ridge-up, film-covering, and double-row planting mode. The specific parameters were: ridge height 10–12 cm, ridge width 80–90 cm; after film covering, plant pineapple seedlings in double rows, row spacing 50–60 cm, plant spacing 35–40 cm, ridge spacing 50–60 cm. At the same time, it suggested that the planting density should be controlled at 45,000 plants per hectare. Excessive density is likely to lead to an increase in small fruits, while being too sparse is not conducive to the growth of pineapple seedlings. The planting depth should be 7–10 cm.

Xie (2019) proposed high-yield cultivation techniques for Tainung 17 pineapples in Changjiang area. The planting density is determined based on various factors such as the variety, soil nutrient conditions and management level. On flat land, it is usually 46,500-49,500 plants per hectare, while on sloping land, it is 42,000-45,000 plants per hectare. The planting methods are divided into two types: One is to plant 4 rows per ridge, with a ridge width of 2.5 meters and a plant spacing of 32–35 cm, using a large and small row interval planting method (large row spacing of 50 cm and small row spacing of 40 cm), which is more suitable for sprinkler irrigation systems; the other is to plant 2 rows per ridge, with a ridge width of 1.3 meters and a plant spacing of 33 cm, which is suitable for combination with drip irrigation systems.

From the existing research and standards, it can be seen that the cultivation mode of pineapples is not closely related to mechanization. When choosing different planting parameters, usually only the simple pineapple agronomic management perspective and practical experience are considered, which is difficult to meet the new requirements of full mechanization for modern pineapple production. Therefore, this paper proposes a double-row staggered planting mode. The specific parameters are: ridge height 20–25 cm, ridge surface width 70 cm, row spacing 35–50 cm, plant spacing 30 cm, ridge spacing 90 cm, planting density 43,500-46,500 plants per hectare, and planting depth 10–12 cm. Through designing different initial positions of the planting devices on both sides, the staggered planting can be achieved. And by adopting the integrated mode of ridge formation, film covering, and double-row planting, the land utilization rate and planting flexibility can be improved, promoting the good growth of pineapple plants and fruits, and laying a good dimensional data foundation for the subsequent design of pineapple harvesting machines.

### 2.2 Measurement of the physical characteristics of large pineapple seedlings

The objects that the pineapple seedling transplanting machine transplants are pineapple seedlings. Pineapple seedling cultivation is an asexual reproduction crop, mainly through nutritional bodies such as crown buds, lateral buds, adventitious buds and stem buds to generate adventitious buds (Rural New Technologies, 2022). When the adventitious buds grow to a certain height, they are used as the seedling materials for reproducing new plants. The pineapple seedlings are different from the slender seedlings of other vegetables. The pineapple seedlings cultivated in China have leaves distributed in a lotus-shaped pattern (Xue et al., 2022).

**Figure 1:**
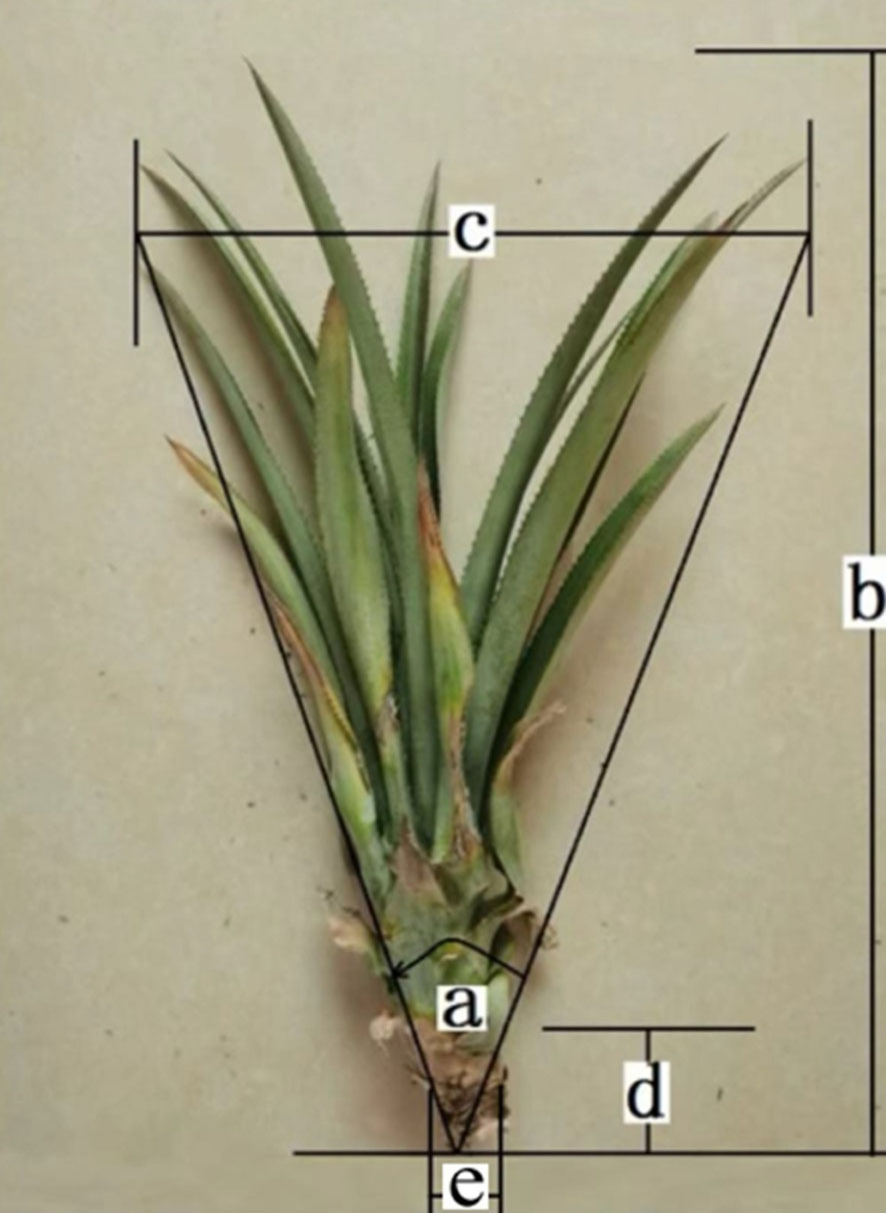
Physical characteristics of large pineapple seedlings. **(a)** represents the cone angle of the plant type, **(b)** represents the plant length, **(c)** represents the plant diameter, **(d)** represents the root length, and **(e)** represents the root diameter.

The pineapple belongs to the Bromeliaceae family and the Bromelia genus. Currently, there are approximately 60 to 70 cultivated varieties. They are generally classified into four categories: Queen (typically from Bali), Kain, Spanish, and Hybrid (typically from Tainung) (Yuan et al., 2020). Therefore, we selected the main cultivated varieties, such as Bali, Golden Pineapple, and Tainung 17, and took 30 plants of each as the research objects. We brought the collected pineapple seedlings back to the laboratory, placed them on the test bench one by one, weighed them, numbered them, and took photos. We used a ruler tool to manually measure the images and recorded the morphological size data. The physical characteristics of the pineapple seedlings are shown in **Figure 1**, where parameter a represents the cone angle of the plant type, b represents the plant length, c represents the plant diameter, d represents the root length, and e represents the root diameter (Xu, 2019).

Statistical analysis was conducted on the morphological data of the measurements, as shown in **Table 1**. It can be observed that there are significant differences among different varieties of pineapple seedlings. The length ranges from 37.00 to 82.80 cm, and the diameter ranges from 12.30 to 63.50 cm.

**Table 1:** Morphological parameters of large pineapple seedlings.

Parameter		Variety		
		Bali	Golden pineapple	Tainung 17
Cone angle of the plant type	Maximum value/cm	74.41	61.82	53.92
	Minimum value/cm	31.21	24.2	25.73
	Mean value/cm	45.39	43.97	41.01
	Coefficient of variation/%	0.22	0.22	0.15
Plant length	Maximum value/cm	61.40	82.80	69.80
	Minimum value/cm	37.00	41.20	41.90
	Mean value/cm	46.61	55.07	53.63
	Coefficient of variation/%	0.13	0.18	0.11
Plant diameter	Maximum value/cm	51.00	63.50	54.80
	Minimum value/cm	26.70	12.30	20.80
	Mean value/cm	35.56	34.91	33.07
	Coefficient of variation/%	0.18	0.39	0.23
Root length	Maximum value/cm	6.90	7.80	8.20
	Minimum value/cm	2.30	2.50	3.10
	Mean value/cm	5.04	4.33	5.23
	Coefficient of variation/%	0.23	0.26	0.21
Root diameter	Maximum value/cm	6.10	5.80	5.60
	Minimum value/cm	2.80	2.70	3.00
	Mean value/cm	4.28	4.14	4.46
	Coefficient of variation/%	0.17	0.19	0.15
Plant weight	Maximum value/cm	392.59	743.19	683.90
	Minimum value/cm	124.78	97.28	141.26
	Mean value/cm	224.13	263.70	317.81
	Coefficient of variation/%	0.29	0.50	0.37

### 2.3 Standardized processing of large pineapple seedlings

**Figure 2:**
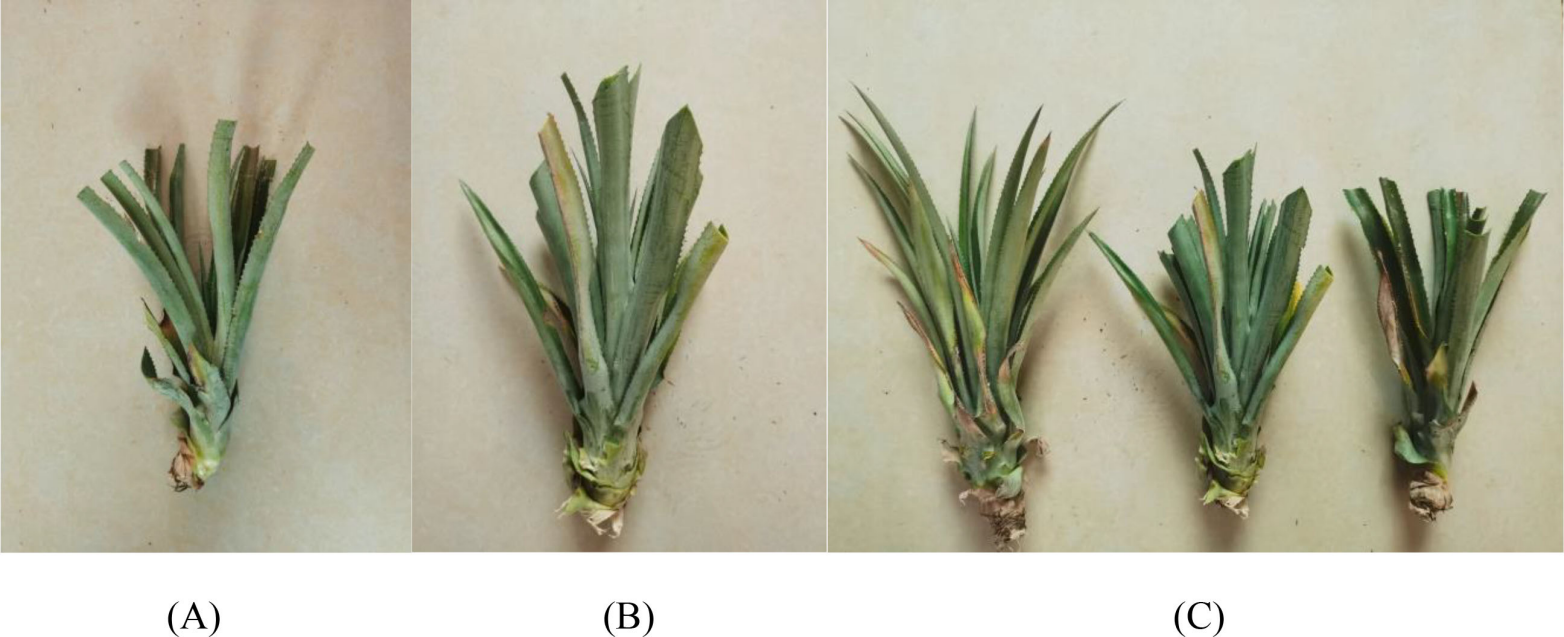
Illustrations of Pruning large pineapple seedlings. **(A)** Horizontal trimming effect. **(B)** 45° trimming effect. **(C)** Trimming effect comparison chart.

To adapt to mechanical planting and prevent the duckbill transplanting device from clamping the extended leaves of large pineapple seedlings during the planting operation when closing and retracting, which would affect the stability and verticality of the pineapple seedlings during transplantation and lead to poor final planting results, it is necessary to standardize the large pineapple seedlings. The standardized length is 15 to 30 cm and the diameter is 10 to 15 cm. Regarding how to carry out the standardization process, this study proposes two standard pruning methods for large pineapple seedlings. The first is the horizontal pruning method, which involves making a horizontal cut at the top of the large pineapple seedling according to the standardized length and diameter requirements. The effect of horizontal pruning is shown in **Figure 2A**, and this method is relatively simple to operate. The second method is the 45-degree pruning technique. According to the standardized length and diameter requirements, two cuts are made at a 45-degree angle at the top of the pineapple seedling. The effect of 45° pruning is shown in **Figure 2B**. This method is more complex than the first one, but it has a better anti-clamping effect. The comparison of the effects of large pineapple seedlings that have not been pruned and those pruned by the two methods is shown in **Figure 2C**.

## 3 Overall structure and working principle

### 3.1 The entire machine structure

**Figure 3:**
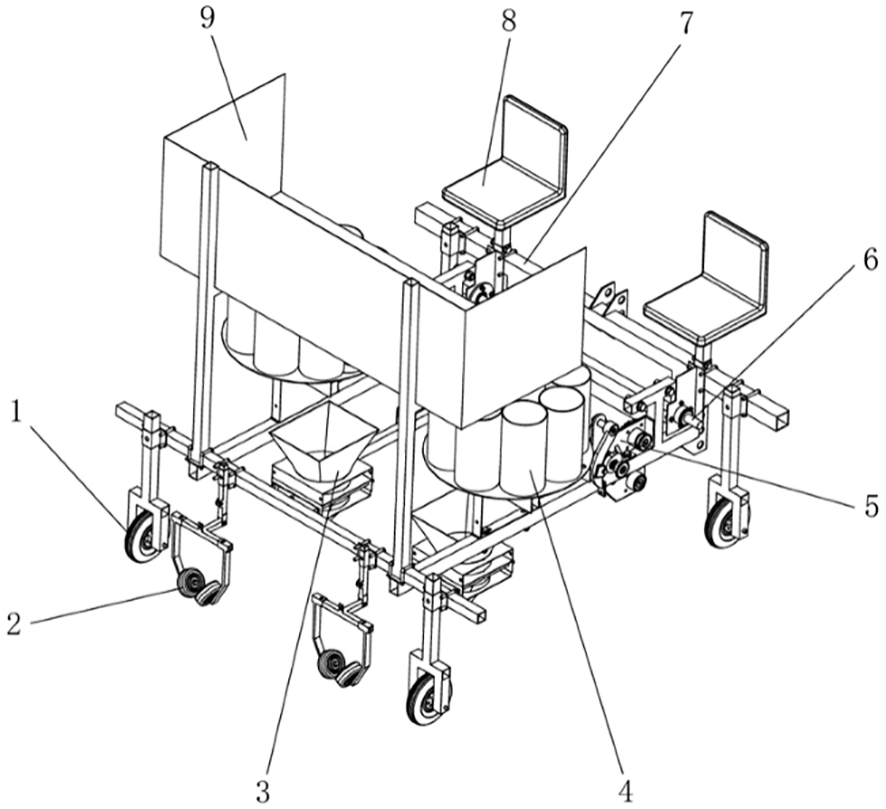
Overall structure of pineapple seedling transplanter. 1. Seed box; 2. Seat; 3. Frame; 4. Drive shaft; 5. Transplanting arm mechanism; 6. Seed cup;7. Seed guiding funnel and duckbill planting device; 8. Soil covering and pressing wheel; 9. Guidance wheel.

The pineapple seedling transplanting machine is mainly composed of transplanting arm mechanism, seed guiding funnel and duckbill planting device, soil covering and pressing wheel, seed box, seed cup, drive shaft, vehicle seat, guide wheel, etc. The transplanting machine is driven by a tractor with a power of 66.19kW or above, and it adopts a three-point suspension system. The power is provided by the ground wheels, and the power is transmitted through chain drive, gearbox and straight gear to be distributed separately to the rotary seed splitting device and planting mechanism, ensuring that the machine transplants seeds while moving, stops transplanting when it stops, and reducing repeated transplanting (Li, 2016). Its overall structure is shown in **Figure 3**.

### 3.2 Design parameter

The pineapple seedling transplanting machine is designed for double-row transplanting. The main design parameters are shown in **Table 2**. The designed pineapple seedling transplanting machine is applicable to farms, professional households and individual farmers. Its total length is 1.8m, width is 1.7m, height is 1.7m, and total weight is 800kg. Therefore, a tractor with a power of 66.19kW or above is selected as the traction power.

**Table 2:** Main design parameters.

Parameter indicators/units	Number
Matching power/kw	>66.19
Number of work rows/rows	2
Overall machine size/(mm ×mm ×mm)	1800 ×1700 ×1700
Capacity of the seed box/L	480
Soil ridge height/mm	200-250
Furrow width/mm	700
Planting row spacing/mm	300
Planting plant spacing/mm	350-500
Planting depth/mm	100-120
Film covering width/mm	1200
Diameter of the seed cup/mm	160
Furrow width/mm	900
Planting frequency/plant/min	120
Transplanting machine operation speed/m/min	36

### 3.3 Operating principle

**Figure 4:**
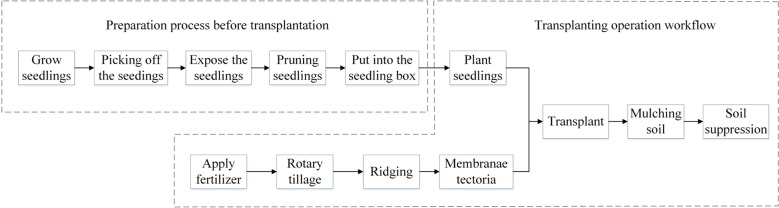
Working process of the transplanter.

The transplanting machine is pulled forward by a tractor, and the traction force is transmitted to the connecting rod mechanism of the transplanting arm. Among them, the sprocket drives the rotating seed cup to rotate, and the operator places the seedlings into the seed cup. The seedlings rotate along with the seed cup and fall into the seed guiding funnel. At the same time, the transplanting arm drives the movement of the seed guiding funnel and the duckbill planting device, achieving the up and down movement of the duckbill device. The opening and closing of the duckbill is controlled by the cam mechanism. When it drops, it opens and plants the pineapple seedlings onto the ridge. Then, it is pressed down by the compaction wheel to complete the transplanting operation. The process of transplanting pineapples is shown in **Figure 4**.

## 4 Design of key components of the transplanting machine

### 4.1 Transmission mechanism design

**Figure 5:**
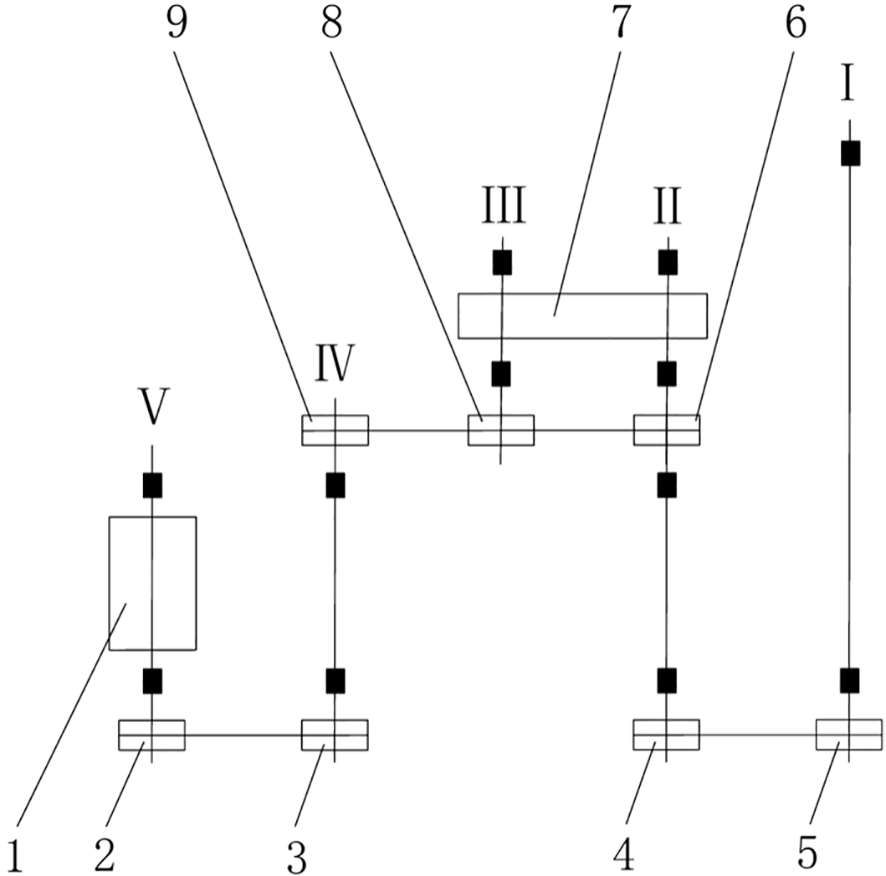
Transplanter transmission mechanism diagram. 1. Seed cup; 2. Sprocket Z_5_; 3. Sprocket Z_4_; 4. Transplanting arm;5. Sprocket Z_3_; 6. Sprocket Z_1_; 7. Sprocket Z_2_; 8. Sprocket Z_6_; 9. Sprocket Z_7._. The Roman numerals are explanatory notes for the components.

The power transmission system of the transplanting machine needs to ensure that the forward speed of the wheels, the swinging speed of the transplanting arm, and the rotation speed of the seed cup are all in harmony during the design. The schematic diagram of the transmission mechanism designed based on actual working conditions is shown in **Figure 5**. This transmission mechanism consists of two parts: the first part is the swinging part of the transplanting arm, where the tractor transmits power to the transplanting arm through chain drive, and the duckbill device is raised and lowered by the swinging of the transplanting arm to achieve the transplanting action; the second part is the rotation part of the seed cup, where the power is transmitted to the seed cup through chain drive, and the seedlings are transported by the rotation of the seed cup.

This transmission system adopts a standardized sprocket configuration. The number of teeth for sprockets Z_1_, Z_6_, and Z_7_ is set to 17 each, while the number of teeth for sprockets Z_2_, Z_3_, and Z_5_ is 38 each. In actual operation, by replacing sprocket Z_2_ and Z_6_, the transmission ratio parameters of the transmission system can be adjusted to meet the differentiated requirements for transplanting operation speed and planting density and other process parameters under different planting conditions (Jin et al., 2019).

### 4.2 Chain drive design

Chain drive is mainly used in places where reliable operation, low-speed heavy-load operation, and harsh working conditions are required (Wang, 2024). Since transplanting machines operate in the field and the environment is harsh, and reliable power transmission is needed, chain drive is chosen as the transmission method for transplanting machines. The following is the design process of the entire chain drive.

1) Select the number of teeth Z_1_ and Z_2_ for the sprocket and determine the transmission ratio iChoose Z_1_ = 17 and Z_2_ = 38, and the transmission ratio is expressed as Equation 1:

(1) $$i=Z_{1}/Z_{2}=2.235$$

2) Determine the calculated power $P_{ca}$Based on the working condition of the chain drive, the number of teeth of the driving sprocket and the number of chain runs, the power transmitted by the chain drive is corrected to the equivalent calculated power of a single-row chain. The calculated power is given by Equation 2:

(2) $$P_{ca}=K_{A}*K_{Z}*P/K_{p}=2.254\left(kw\right)$$

In the formula, $K_{A}$ is the operating condition coefficient, referring to “Mechanical Design” (Pu et al., 2019), and it is set to 1; $K_{A}$ is the coefficient of the active sprocket teeth, $K_{Z}$ = (19/Z_1_)1.08 = 1.127; $K_{p}$ is the multi-row chain coefficient, set to 1; P is the transmitted power, set to 2 kw.

3) Determine the chain model and pitch P

Set the tractor operation speed to 0.6 m/s, n_2_ = 120 r/min. Based on the transmission ratio, n_1_ = 268 r/min can be obtained.

According to the calculated power $P_{ca}$ of the equivalent single-row chain, the rated power $P_{c}$ of the single-row chain, and the rotational speed n_1_ of the driving sprocket, it can be found from “Mechanical Design” that the chain model of 08A can be selected. The chain pitch P = 12.70 mm is determined.

4) Calculate the number of links and the center distance

The initial center distance $a_{0}$ is set at 30P = 381mm, and the corresponding number of link sections as Equation 3:

(3) $$L_{P0}=2\frac{a_{0}}{p}+\frac{z_{1}+z_{2}}{2}+{\left(\frac{z_{2-}z_{1}}{2\Pi }\right)}^{2}\frac{p}{a_{0}}=89.72$$

To avoid using transitional links, the calculated link number L_P0_is rounded to an even number, and L_P_ = 88 is taken. From “Mechanical Design”, it can be found that the calculation coefficient for the center distance f_1_ = 0.248. Then, the maximum center distance of the chain drive is given by Equation 4

(4) $$a_{max}=f_{1}p\left[2L_{p}-\left(Z_{1}+Z_{2}\right)\right]=371\left(\text{mm}\right)$$

5) Main Design Conclusions

Chain model 08A; Number of chain teeth Z_1_ = 17, Z_2_ = 38; Number of chain links L_P_ = 88; Center distance a= 371mm.

### 4.3 Structural design of planting device and connecting rod mechanism

The performance of the planting equipment is the key factor restricting the development of the machinery for planting on membranes. Currently, the planting techniques abroad are relatively mature, but the equipment developed is mostly large-scale planting machinery, which is not only expensive but also difficult to meet the actual needs of China. In contrast, the research in the field of planting machines in China started relatively late, especially the exploration of planting machines on membranes is still insufficient. In the membrane planting operation, the existence of problems such as scraping the membrane and tearing the membrane further hinders the promotion and application of China’s membrane planting machines (Li, 2016).

During the design process of the planting device and the linkage mechanism, it is necessary to plan out an ideal planting trajectory based on agronomic requirements and the operational characteristics of the transplanting machine. This approach helps to reduce the deviation between the actual movement trajectory of the transplanting mechanism and the ideal trajectory, thereby improving the accuracy and efficiency of the design.

In the field of mechanism motion synthesis, research on linkage mechanisms has been quite extensive. Suh and Radcliffe (1967) used matrix methods to construct a series of nonlinear equation systems to solve the precise synthesis problems of planar four-bar and five-bar mechanisms, achieving the precise synthesis of up to five positions. Burmester (1888) began from a purely geometric perspective and explored the precise synthesis methods of planar linkage mechanisms in 3, 4, and 5 positions. Liu and Yang (1999) proposed a two-level optimization strategy based on the continuous optimization method. Zhao et al. (2016) conducted precise, approximate, and mixed synthesis research on multiple positions of planar four-bar mechanisms using the motion mapping method. Han and Qian (2009) proposed the concepts of mapping and solution domains, visually presenting the different performance attributes, distribution, and change trends of the mechanism within a finite solution domain based on 4 positions, and then constructing the feasible solution domain of the mechanism by applying constraint conditions to complete the mechanism screening. This concept was also applied to the research on the generation of various motions of linkage mechanisms.

**Figure 6:**
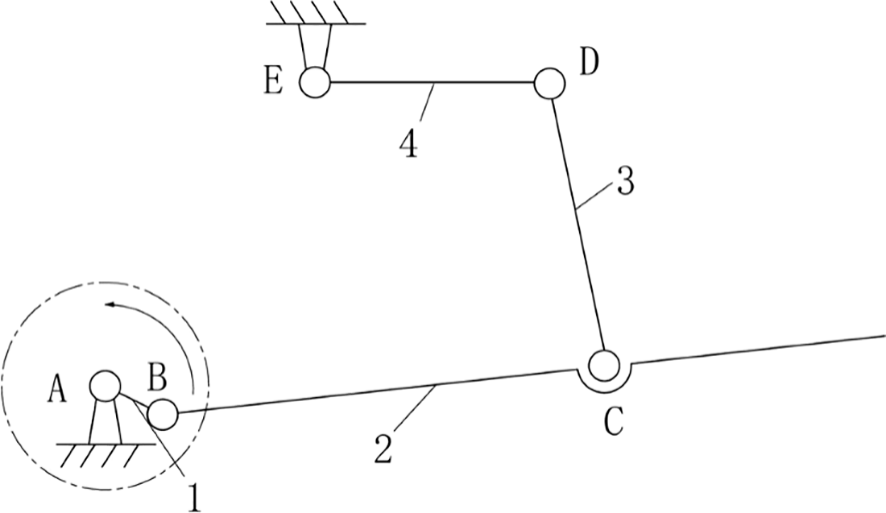
Motion diagram of connecting rod mechanism.

Based on the above content and in combination with the transmission mechanism and chain drive design, this paper has designed a connecting rod planting mechanism, integrating and simplifying the two functions of the planting device’s up-and-down movement in the pineapple seedling transplanting machine. This enables the duckbill planting device in the transplanting machine to complete the planting trajectory and posture on the film, improving the uprightness of the pineapple seedlings, achieving stable planting depth, without damaging the seedlings, and increasing the survival rate of the seedlings, ensuring that the pineapple seedlings are accurately transplanted to the field at the prescribed row spacing and plant spacing (Jin et al., 2016; Huang, 2016; Yang, 2019). The kinematic sketch is shown in **Figure 6**.

### 4.4 Overall assembly of the machine

**Figure 7:**
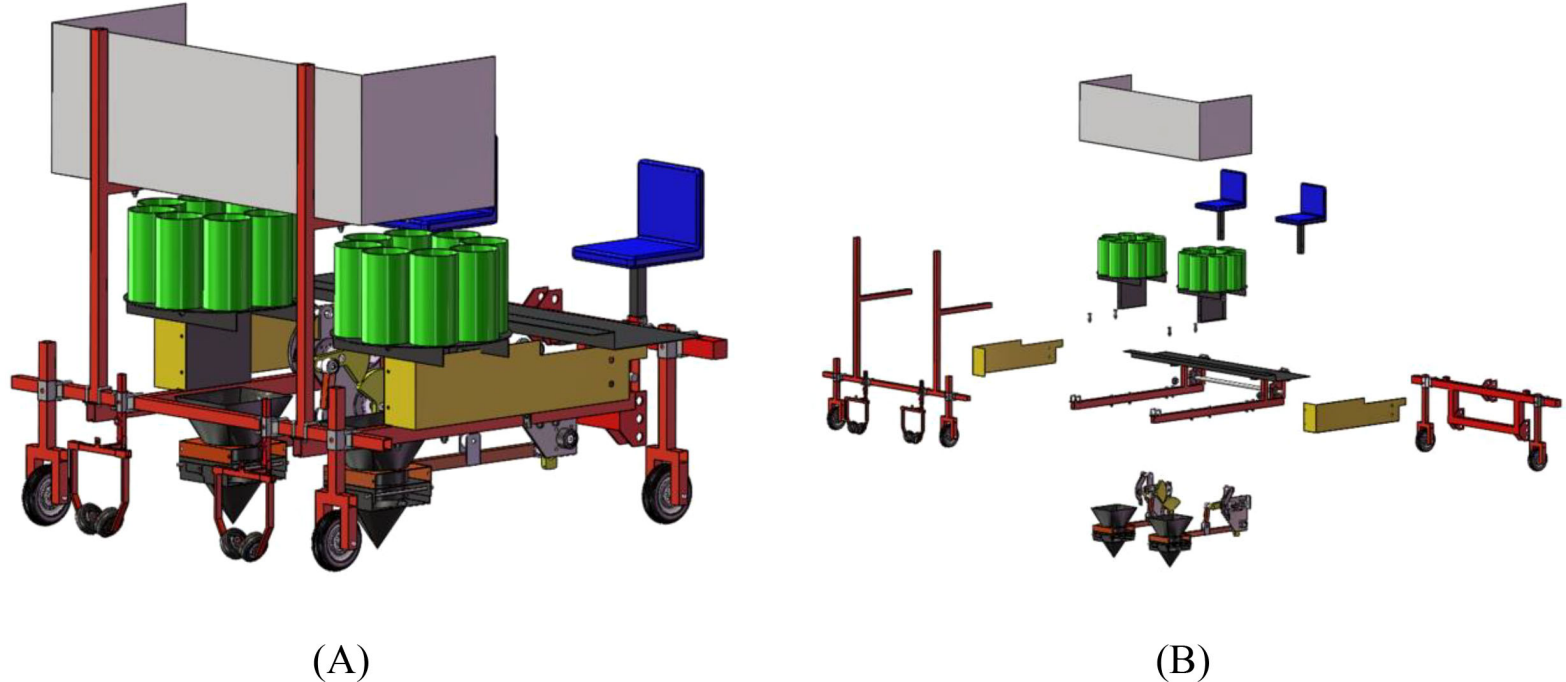
Three-dimensional modeling of pineapple seedling transplanting machine. **(A)** Assembly simulation diagram. **(B)** Simulated explosion pattern.

Based on the agronomic cultivation requirements of pineapple seedlings and the working principle of the duckbill transplanting machine (Cui et al., 2023), the dimensions of each component were designed. The three-dimensional modeling and overall assembly of the machine were carried out using the SolidWorks software, and the interference detection function of assembly components in the software was utilized to confirm that the three-dimensional assembly model had no interference. From the very beginning of the design process, ensure that the machines used in the subsequent actual production can stably achieve all the preset functions. The 3D modeling of the pineapple seedling transplanter is shown in **Figure 7**, where the assembly simulation is presented in **Figure 7A** and the simulation explosion view is shown in **Figure 7B**.

## 5 Finite element analysis of the main load-bearing components of the transplanting machine

The main body of the pineapple seedling transplanting machine serves as the “ skelecton” of the equipment, and needs to bear components such as the transplanting mechanical arm and the power system. It also operates in harsh conditions in southern fields, including high temperatures, high humidity, frequent bumps and impacts, and exposure to pesticides in the soil. Therefore, by comprehensively analyzing the four main requirements of reliable load-bearing capacity, anti-shock fatigue strength, environmental corrosion resistance, and cost controllability. A medium-carbon alloy structural steel of model 40CrNiMoA and normalized treatment was selected as the material for the main load-bearing components of the transplanting machine. Compared with the commonly used 45 steel in its normalized state, its yield strength increased by 135%, tensile strength increased by 33%, and fatigue strength increased by 40%. It can stably bear the weight of the components and the instantaneous inertia force, traction overload force, etc. of the transplanting process, meeting the fatigue life requirements for frequent field operations of the transplanting machine, avoiding frame bending and deformation (such as the descending of the transplanting arm caused the deviation of the pineapple seedlings during transportation), and ensuring the accuracy and verticality of the transplanted seedlings.

During the simulation process, pressure is applied at the key stressed points to simulate the forces acting under the working conditions. The simulation mainly uses the SolidWorks software to conduct finite element analysis on the main load-bearing components of the pineapple seedling transplanting machine. The specific analysis is as follows:

### 5.1 Rack

**Figure 8:**
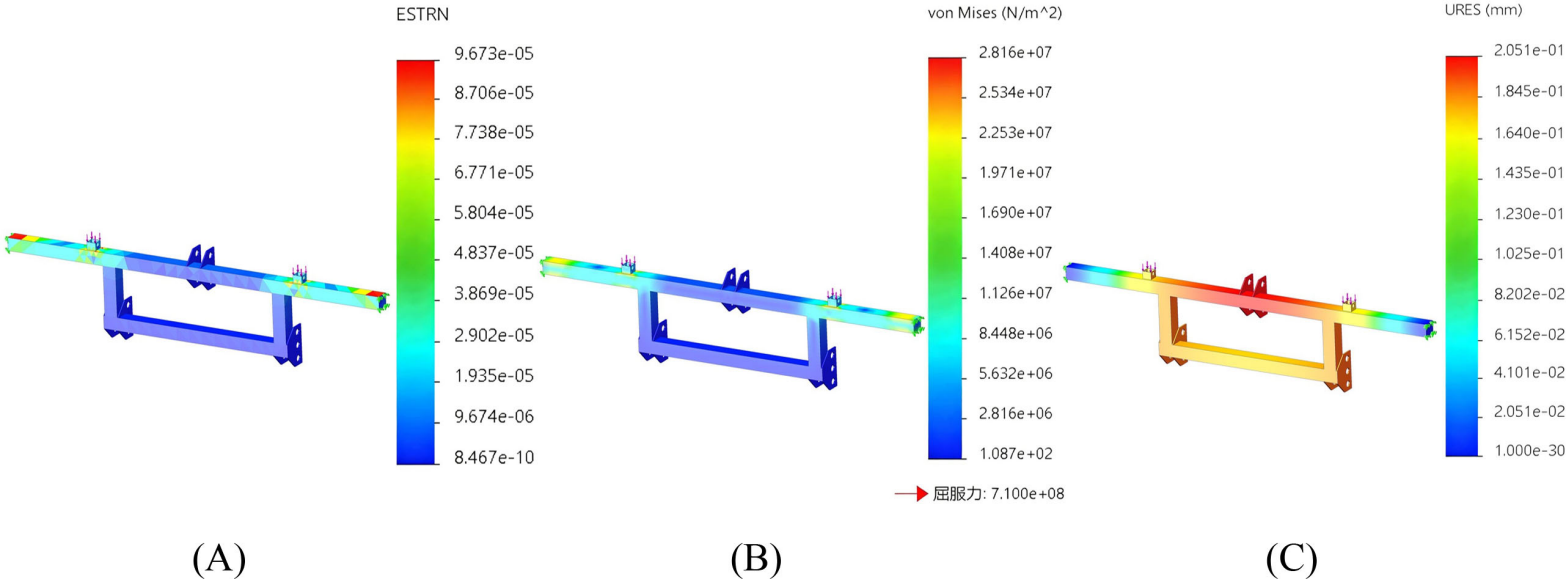
Finite element analysis of the rack. **(A)** Finite element analysis strain figure of the rack. **(B)** Finite element analysis stress diagram of the rack. **(C)** Finite element analysis displacement diagram of the rack.

Both ends of the frame are connected to other parts of the frame. To simulate the rigid support of the frame by the frame body, fixed constraints are set at both ends. A load of 2000N is applied at the connection between the seat and the frame body (including the seat weighing 20kg, two adult laborers weighing 75kg each, and other parts weighing 30kg). The seedling box and the rotary seedling cup are installed on other frames, and here the frame under the seat is taken as an example. The finite element analysis results of the strain, stress and displacement of the frame are shown in **Figure 8A**, **Figure 8B** and **Figure 8C** respectively.

The strain, stress and displacement diagrams obtained from the finite element analysis of the frame show that the maximum strain is 9.673e-05, the maximum stress is 2.816e+07 Pa, and the maximum displacement is 2.051e-01 mm. As can be seen from **Figure 8B**, the yield force of the frame is approximately 282 MPa. The stress it bears does not exceed the material’s yield strength of 710 MPa, which meets the design requirements.

### 5.2 Driving shaft

**Figure 9:**
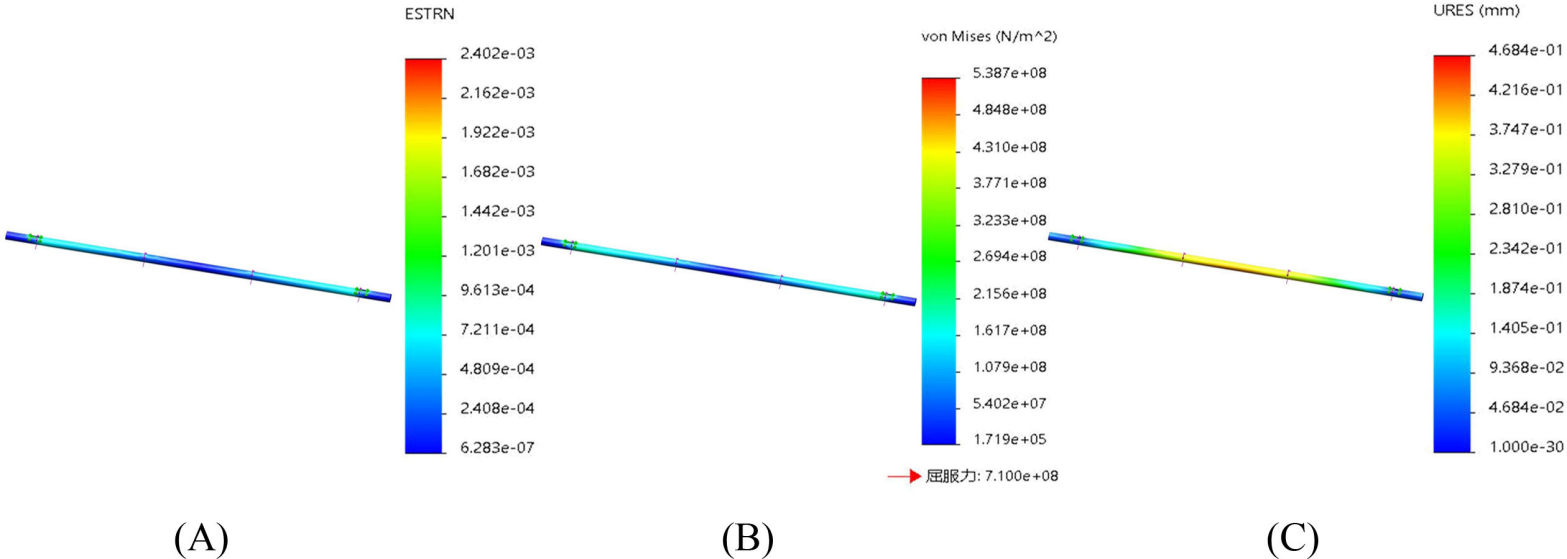
Finite element analysis of the driving shaft. **(A)** Finite element analysis strain figure of the driving shaft. **(B)** Finite element analysis stress diagram of the driving shaft. **(C)** Finite element analysis displacement diagram of the driving shaft.

The driving shaft, as a transmission component, mainly bears the torque force transmitted to the rotary seedling cup. The driving shaft is connected to the frame through rolling bearings, so cylindrical surface fixed constraints are set at the bearing installation parts at both ends. The torque load is applied to the keyway boundary and the model surface of the shaft. The load is set at 1500N (According to the torque formula T = 9550×P/n=F×d/2, where P = 2.1kw, n=268 r/min, and d=50 mm, it is calculated that the force that the driving shaft needs to bear is approximately 3000 N, and the force on one side is 1500 N). The finite element analysis results of strain, stress, and displacement of the driving shaft are shown in **Figures 9A–C** respectively.

From **Figure 9**, the maximum strain is 2.402e-03, the maximum stress is 5.387e+08 Pa, and the maximum displacement is 4.684e-01 mm. According to **Figure 9B**, the yield force of the active shaft is around 539 MPa, and the yield strength of the used material is 710 MPa, which fully meets the design requirements.

### 5.3 Transplanting arm

**Figure 10:**
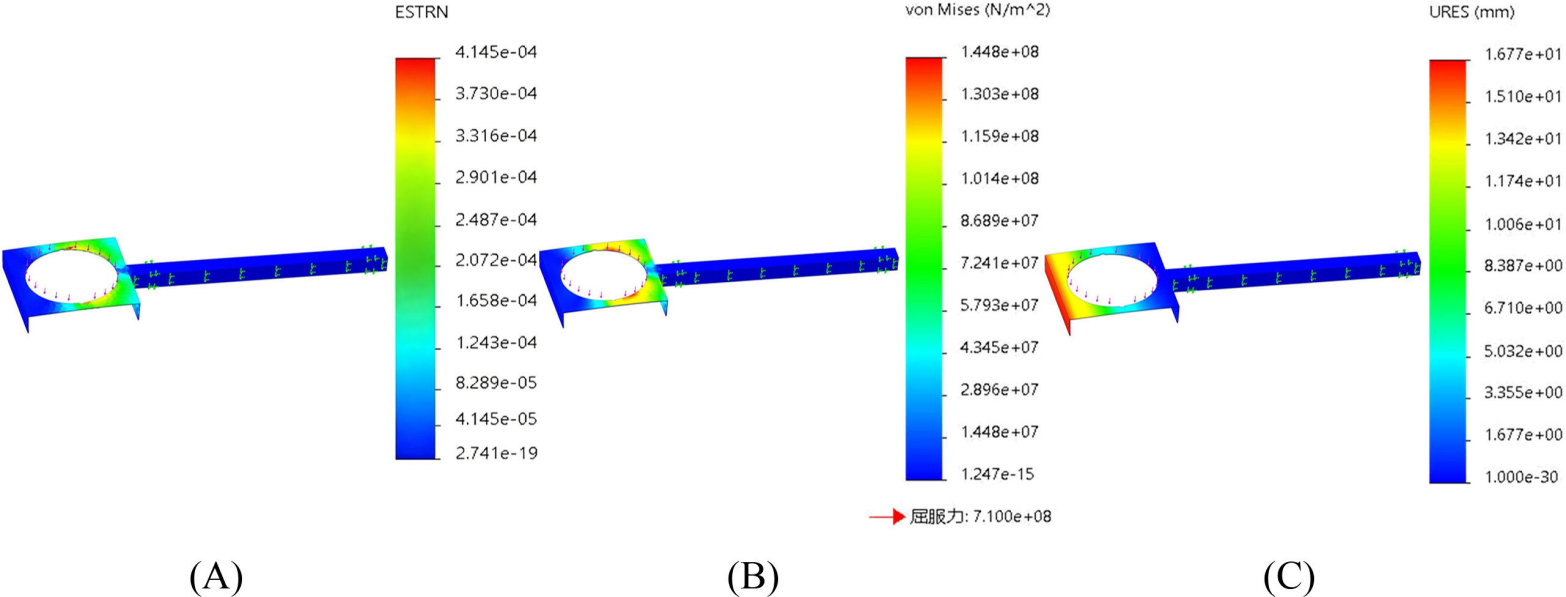
Finite element analysis of the transplanting arm. **(A)** Finite element analysis strain figure of the transplanting arm. **(B)** Finite element analysis stress diagram of the transplanting arm. **(C)** Finite element analysis displacement diagram of the transplanting arm.

The transplanting arm is connected to the connecting rod mechanism and the seedling guide device, and operates through chain drive. A rigid fixed constraint is set at the fixed end of the connecting rod mechanism, and the free end is connected to the seedling guide device, which is in an suspended state and is only subjected to load. A force of 100N is applied to the boundary between the transplanting arm and the seedling guide device (including the 6kg of the seedling guide device, 3.75kg of the duckbill device, and 0.25kg of the pineapple seedlings). The finite element analysis results of the strain, stress and displacement of the active shaft are shown in **Figure 10A–C** respectively.

From **Figure 10**, the maximum strain is 4.145e-04, the maximum stress is 1.448e+08 Pa, and the maximum displacement is 1.677e+01 mm. The structural design and material selection of the transplanting arm meet the requirements.

In conclusion, through the finite element analysis of the main load-bearing components of the pineapple seedling transplanting machine, the simulation analysis can effectively reduce the initial design cost, verify the correctness of the structure and material selection of the pineapple seedling transplanting machine, and ensure the stability of the component performance.

## 6 Field experiment

The field experiment was conducted on August 23, 2025, at the pineapple planting experimental base of Nuoxiangyuan Agricultural Products Cooperative in Qujie Town, Xuwen County, Guangdong Province, China. The experimental equipment was the pineapple seedling transplanter developed for this research. The experimental material was the Bali variety of pineapple. The experimental area was approximately 667 m^2^, and the planting density of large pineapple seedlings within the area was 2,900 to 3,100 plants per 667 m^2^.

**Figure 11:**
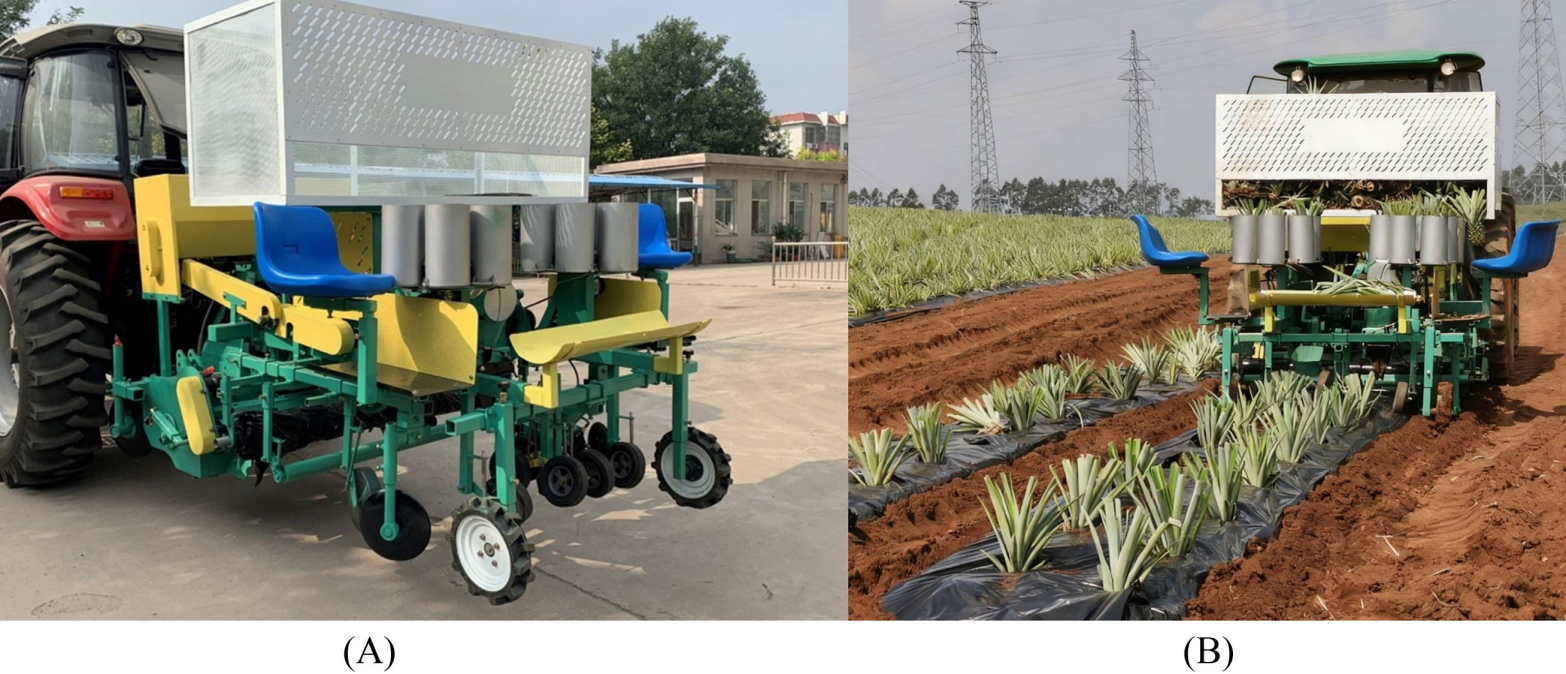
Field test of the pineapple seedling transplanting machine. **(A)** Prototype drawing of pineapple seedling transplanting machine. **(B)** Pineapple seedling transplanting machine transplants pineapple seedlings.

The prototype diagram of the pineapple seedling transplanting machine is shown in **Figure 11A**. During the production process of the pineapple seedling transplanter, certain adjustments and optimizations will be made to the components based on actual conditions, such as the position of the seat and the shape of the seedling box. However, the core key components remain unchanged and do not affect the function of the transplanter.

The seedlings are placed by a horizontal seeding device in the transplanting machine, ensuring sufficient time for the placement process, thereby effectively improving the transplanting efficiency. The size of the seedling box is 1.6 meters in length, 0.5 meters in width, and 0.6 meters in height, with a volume of 480 liters. According to the standardized size of pineapple seedlings (length 15–30 centimeters, diameter 10–15 centimeters), and considering that the pineapple seedlings placed horizontally in the seedling box will be subjected to the pressure from the upper seedlings, one seedling box can accommodate approximately 600 seedlings. Taking the Bali variety as an example, each individual seedling weighs about 250 grams, so one seedling box can carry approximately 300 kilograms of large pineapple seedlings. In the planting area, if large pineapple seedlings are planted at a density of 2,900-3,100 plants per 667 square meters, then 5 seedling loading operations are required for each 667 square meters.

In the pineapple transplanting operation, each field ridge is approximately 60 meters long, and multiple stops are required for supplying. When manually retrieving seedlings, workers need to travel to both ends of the ridge and carry the seedlings between the ridges when returning. Currently, there is a lack of specialized vehicles for transporting seedlings across ridges, which means that each time seedlings need to be replenished, the transplanting operation has to be interrupted. A significant amount of time is wasted on retrieving, transporting, and stopping to replenish seedlings. Additionally, after the transplanting machine reaches the end of a ridge, it needs to turn around and align with the next ridge to continue the operation. According to actual operation statistics, an average of 12 turns are required for every 667 square meters of field. Frequent turning further reduces the effective transplanting time and lowers the area covered per unit time. The time spent on auxiliary operations (stopping to load seedlings and turning the machine) accounts for a large proportion of the total operation time, which not only severely restricts the overall work efficiency but also significantly increases the labor intensity of workers. To address these issues, this article presents an optimized plan for placing seedlings at fixed points in the field. At each end of each ridge, 400 pineapple seedlings are pre-positioned, matching the seedling requirement for completing a single ridge (400 seedlings). The seedling loading process does not require workers to enter the ridges; instead, ordinary transport vehicles can directly deliver the seedlings to both ends of the ridges, eliminating the need for cross-ridge transportation. At the same time, the turning operation of the transplanting machine is synchronized with the process of replenishing the seedlings. The seedling box capacity of the transplanting machine is 600 seedlings. When the machine is first operated, the seedling box is filled. After completing the planting of one ridge, while the machine is turning and adjusting its direction, seedlings can be quickly replenished from the pre-positioned seedlings at the end of the ridge to 600 seedlings, achieving “no waiting during turning and no additional stops for seedling replenishment”. Although it is still rather troublesome, it does improve the overall efficiency of pineapple transplantation to a certain extent.

The field experiments revealed that in terms of manual planting, 10 people could complete approximately 20,000 square meters in a day, which means each person could plant about 2,000 square meters per day. In terms of mechanical planting, 4 people working in conjunction with machinery could complete approximately 1,334 square meters per hour. Calculated based on an 8-hour work schedule, they could complete approximately 10,000 square meters in a day, equivalent to about 2,500 square meters per person per day. However, the time spent on mechanical operations was mainly wasted in turning around, loading seedlings, and other processes. Due to the large size of the pineapple seedlings, the loading capacity is limited, and they cannot be sown directly like the seeders used for planting corn or wheat. Therefore, the mechanization of transplanting has a slight improvement in efficiency, mainly by greatly reducing labor intensity and labor costs. The pineapple seedling transplanting machine transplants pineapple seedlings as shown in **Figure 11B**. The comparison of data between manual and machine transplanting is presented in **Table 3**.

**Table 3:** Comparison of manual and transplanter planting data.

Data	Traditional manual planting method	Mechanized planting methods
Number of plants	4000 plants per 667 square meters	3000 plants per 667 square meters
Labor cost	Approximately $42 per person	Approximately $14 per person
Efficiency	20,000 square meters per day	10,000 square meters per day
Supporting power	>29.42kW	>66.19kW
Number of employees	>10 people	>4 people
Top application	Artificial fertilization	Machine fertilization/Foliar fertilizer
Field management	Artificial pest control	Agricultural machinery spraying

## 7 Conclusion

To ensure the efficient operation of the pineapple seedling transplanting machine, structural design and size parameter optimization were carried out. The specific research results are as follows:1) A comprehensive analysis of the planting mode and physical characteristics of pineapple seedlings was conducted, providing a reference basis for the design and development of the pineapple seedling transplanting machine and key planting components.2) The transmission system of the optimized transplanting machine was designed and calculated. The chain transmission design results: the chain model is 08A, the number of teeth Z_1_ = 17, Z_2_ = 38, the number of links L_P_ = 88, and the center distance a = 371mm. This provides reliable power transmission for the transplanting machine, ensuring that the forward speed of the wheels, the swing speed of the transplanting arm, and the rotation speed of the seed cup are matched, ensuring that the machine transplants seedlings while moving, stops while stopping, and reduces repeated transplanting.3) The entire machine was modeled in three dimensions using SolidWorks. By utilizing the interference detection function of the assembly components in this software, it was confirmed that there was no interference in the assembly model, meeting the design requirements. Additionally, based on the actual force conditions, finite element analysis was conducted on its core components. The analysis results indicated that when applying forces of 2,000N, 1,500N, and 100N respectively to the frame, the active shaft, and the transplant arm, the maximum stress was 2.816e+07 Pa, 5.387e+08 Pa, and 1.448e+08 Pa, respectively, which did not exceed the yield strength of 710 MPa of the used materials. This verified the correctness of the structural design and the safety of the structural strength and deformation of the mechanism.4) The field experiments showed that the transplanting machine for pineapple seedlings achieved good results. In traditional manual planting, each person could plant 2,000 square meters per day, while with mechanized planting, each person could plant 2,500 square meters per day. A field fixed-point planting plan was proposed, realize the ability of the transplanting machine to turn around while supplying the seedlings. The transplanting efficiency has been significantly improved. What’s more, it effectively alleviated the labor intensity and reduced the labor costs.

In summary, by using computers to complete the digital design of machines, the design rationality is fully verified before starting actual production. This helps reduce production losses caused by design flaws at the source and provides positive reference for the design and production of similar machines in the future. Based on this, in the future, we can focus on deepening technology and expanding functions, and promote the improvement and research of transplanting machine design from multiple dimensions. The specific directions are as follows: 1) Integrate digital twin technology to build a dynamic model of the transplanting machine, realize online monitoring of its working state and real-time assessment of transplanting effects, accurately simulate different varieties of pineapple seedlings and complex field conditions in southern areas, and improve the adaptability of the equipment to diverse conditions. 2) By integrating GPS positioning and IoT modules, real-time data such as the number of transplanted plants in the field, operation speed, and equipment malfunctions are collected. These data are then synchronized and pushed to farmers via a mobile app. This not only enables “traceability of the planting process and analysis of operation efficiency”, but also provides precise data support for subsequent field management tasks such as fertilization and pest control, thus completing the data link from “transplanting to management”.3) Promote the research and development of unmanned operation technology:On the one hand, use GPS navigation technology to achieve automatic path planning for the transplanting machine, reducing manual alignment operations; on the other hand, design automatic seedling extraction and planting components on the transplanting machine, and develop cross-row-specific seedling transportation vehicles to achieve automatic replenishment of pineapple seedlings, solving the problems of manual cross-row transportation and machine stop for seedling replenishment, and achieving full-process unmanned transplanting. 4) Build a metaverse platform, establish a deep coupling model of “mechanism - environment - crop” in the virtual environment, form a dynamic monitoring and optimization system covering the entire transplanting cycle, and provide more efficient virtual testing and verification scenarios for the continuous iteration of transplanting technology. Through these multi-dimensional improvements, we can gradually achieve the upgrade of pineapple transplanting machines from “digital design” to “intelligent operation” and “precision management”, further improving the operation efficiency of the equipment and reducing the reliance on manual labor, and providing more powerful technical support for the large-scale and modernized cultivation of pineapples.

## Data Availability

The raw data supporting the conclusions of this article will be made available by the authors, without undue reservation.

## References

[B1] Burmester L. (1888). Lehrbuch der kinematik. (Leipzig: Verlag Von Arthur Felix Press).

[B2] Chen M. Z. Zheng S. Deng G. R. Cui Z. D. Li L. Qin S. M. (2021). Analysis and prospect of pineapple mechanized production in zhanjiang. Modern Agric. Equipment. 42, 18–21.

[B3] Cui Z. D. Chen M. W. Xie J. Q. Deng G. R. Zhou W. Zheng S. (2022). Preliminary study on the cultivation mode of mechanized pineapple and its mechanization in zhanjiang. Modern Agric. Equipment. 43, 2–9.

[B4] Cui Z. D. He F. G. Li L. L. Jing F. L. Zhang M. Q. Su Z. Q. (2023). Design and test of chain-clamp pineapple transplanting mechanism. Modern Agric. Equipment. 44, 57–64.

[B5] Deng X. F. Yan Q. Chen D. Y. Song H. J. Wu M. C. (2019). Research status and development trend of pineapple picking machine. Forestry Machinery Woodworking Equipment. 47, 4–8. doi: 10.13279/j.cnki.fmwe.2019.0100

[B6] Djido U. Fassinou Hotegni N. V. Lommen W. J. M. Hounhouigan J. D. Achigan-Dako E. G. Struik P. C. Li Y. D. (2021). Effect of planting density and K2O:N ratio on the yield, external quality, and traders’ Perceived shelf life of pineapple fruits in Benin. Front. Plant Sci. 12. doi: 10.3389/FPLS.2021.627808, PMID: 34220877 PMC8244590

[B7] Dong L. Q. Yang T. X. Ma L. Li Y. D. (2025). Optimization of row and hill spacing patterns improved rice population structure and increased rice yield. Front. Plant Sci. 16, 23–30. doi: 10.3389/FPLS.2025.1570845, PMID: 40491825 PMC12146203

[B8] Guo Z. X. Zhang H. (2018). High-yield cultivation techniques for perfume pineapples in changjiang area. China Trop. Agriculture. 5, 78–80.

[B9] Han L. H. Mao H. P. Yan L. Hu J. P. Huang W. Y. Dong L. L. (2015). Pincette-type end-effector using two fingers and four pins for picking up seedlings. Trans. Chin. Soc. Agric. Machinery. 46, 23–30. doi: 10.6041/j.issn.1000-1298.2015.07.004

[B10] Han J. Qian W. (2009). On the solution of region-based planar four-bar motion generation. Mech. Mach. Theory. 44, 457–465. doi: 10.1016/j.mechmachtheory.2008.03.005

[B11] He F. Zhang Q. Deng G. Li G. J. Yan B. Pan D. X. (2024). Research status and development trend of key technologies for pineapple harvesting equipment: A review. Agriculture 14, 975–975. doi: 10.3390/AGRICULTURE14070975

[B12] Huang S. S. (2016). Design and Experimental Study on the Soil Covering System of the Up-Film Transplanting Machine. (dissertation/master's thesis). (China (Shaanxi): University of Northwest Agriculture & Forestry).

[B13] Jia J. (2017). Accelerate the integration of agricultural machinery and agricultural techniques to promote the development of agricultural mechanization. Farm Machinery 4, 75–78. doi: 10.16167/j.cnki.1000-9868.2017.04.014

[B14] Jin X. Du X. W. Yang C. H. Ji J. T. Wang S. G. Yan H. (2016). Design and experiment on crank-chute planting mechanism of transplanting machine. Trans. Chin. Soc. Agric. Machinery. 47, 83–90. doi: 10.6041/j.issn.1000-1298

[B15] Jin X. Li X. Y. Li X. Zhang Y. Z. Guo W. B. Zong Z. Y. (2019). Experimental study on the ratio of the transmission system of the lifting cup transplanter under the characteristics of soil filling and silting in hetao area. J. Agric. Mechanization. 41, 57–61. doi: 10.13427/j.cnki.njyi.2019.11.010

[B16] Kossi E. M. G. Beyegue D. H. Boukong A. Ossogo R. G. Lidjo L. (2025). Optimizing pineapple production under waterlogged soil condition in low input management using adequate ridge tillage height and plant density. Front. Plant Science. 16. doi: 10.3389/FPLS.2025.1570261, PMID: 40530283 PMC12170611

[B17] Li J. (2016). Design and Optimazation of Planting Mechanism. (dissertation/master's thesis). (China (Shaanxi): Northwest Agriculture & Forestry University).

[B18] Li L. L. (2022). Design and Experiment of Chain Clip-Type Pineapple Seeding Transplanting Mechanism Based on Self-Balancing. (dissertation/master's thesis). (China (Wuhan): Huazhong Agricultural University). doi: 10.27158/d.cnki.ghznu.2022.001618

[B19] Liao J. W. Zhu Y. Q. Luo K. Wu W. B. Ma Y. L. Xu P. B. . (2014). Exploring the development of pineapple harvesting mechanization. Modern Agric. Equipment. 5, 56–59.

[B20] Liu C. H. He H. Zhou C. P. Wu X. M. Kuang R. B. Yang M. . (2024). High-quality and high-efficiency cultivation model of “One fertilizer-two prevention-three reduction” for pineapple production. China Trop. Agriculture. 4, 72–75.

[B21] Liu A. X. Yang T. L. (1999). Finding all solutions to unconstrained nonlinear optimization for approximate synthesis of planar linkages using continuation method. J. Mechanical Design. 121, 368–374. doi: 10.1115/1.2829469

[B22] Lyu Z. J. Shan Y. Y. Wang J. Zhao J. (2017). Research progress of vegetable transplanting machine and prospects of seedling-picking machinery of transplanter. J. Chin. Agric. Mechanization. 38, 30–34. doi: 10.13733/j.jcam.issn.2095-5553.2017.11.006

[B23] Office of South Subtropical Crops Development, Ministry of Agriculture (2020). Production of tropical and south subtropical crops in China. Available online at: https://www.catas.cn/EN/contents/1270/39015.html.

[B24] Pu L. G. Chen D. G. Wu L. Y. (2019). Mechanical Design. (Beijing: Higher Education Press).

[B25] Qiu J. Y. Duan J. L. Zhang Z. X. Xue Z. (2025). Parameter design and experiment of rotary plate pineapple fruit picker. Front. Plant Science. 16. doi: 10.3389/FPLS.2025.1575648, PMID: 40487212 PMC12141857

[B26] Rural New Technologies (2022). Pineapple seedling cultivation techniques. 09, 18–19. doi: CNKI:SUN:NCXJ.0.2022-09-033

[B27] Shao Y. Y. Liu Y. Xuan G. T. Gao X. M. Han X. Wang Y. X. (2019). Design and simulation analysis of multi-function duckbill type vegetable transplanter. J. Chin. Agric. Mechanization. 40(11) 9-12, 34. doi: 10.13733/j.jcam.issn.2095-5553.2019.11.02

[B28] Suh C. H. Radcliffe C. W. (1967). Synthesis of plane linkages with use of the displacement matrix. J. Eng. Industry. 89, 215. doi: 10.1115/1.3610030

[B29] Sun W. S. (2020). Introduction and cultivation points of tainong 16 pineapple in zhanjiang, guangdong province. Sci. Cultivation Breeding. 2, 25–26. doi: 10.13270/j.cnki.kxzh.2020.02.010

[B30] Wang Q. Q. (2024). Design and analysis of transmission system of grass grid paving vehicle. Mach. Building Automation. 53, 78–81. doi: 10.19344/j.cnki.issn1671-5276.2024.01.015

[B31] Wen Y. S. Zhang J. X. Tian J. Y. Duan D. S. Zhang Y. Tan Y. Z. . (2021). Design of a traction double-row fully automatic transplanter for vegetable plug seedlings. Comput. Electron. Agric. 182, 106017. doi: 10.1016/J.COMPAG.2021.106017

[B32] Xie C. Q. (2019). High-yield cultivation techniques for pineapple variety no. 17 of Taiwanese farmers in changjiang area. Bull. Agric. Sci. Technology. 12, 312–314.

[B33] Xu G. W. (2019). Key Components Research and Design of Double-row Crossing Salvia Miltiorrhiza Transplanter on Mulch-Film of Big Ridge. (dissertation/master's thesis). (China (Harbin): Northeast Agricultural University).

[B34] Xue Z. Chen R. Y. Zhang X. M. (2021). Frontier of pineapple planting and harvesting mechanization in the world. J. Shanxi Agric. Univ. (Natural Sci. Edition). 41, 110–120. doi: 10.13842/j.cnki.issn1671-8151.202103002

[B35] Xue Z. Zhang X. M. Chen R. Y. Wang S. Pan R. (2022). Design and experiment of 2ZBL-90 double row pineapple planter. J. Chin. Agric. Mechanization. 43, 9–14. doi: 10.13733/j.jcam.issn.2095-5553.2022.06.002

[B36] Yang W. B. (2019). Research and Development of key Components of Tobacco Seedling Transplanting Machine. (dissertation/master's thesis). (China (Kunming): Kunming University of Science and Technology). doi: 10.27200/d.cnki.gkmlu.2019.000005

[B37] Yu G. H. Wang L. Sun L. Zhao X. Ye B. L. (2022). Advancement of mechanized transplanting technology and equipments for field crops. Trans. Chin. Soc. Agric. Machinery. 53, 1–20. doi: 10.6041/j.issn.1000-1298.2022.09.001

[B38] Yuan J. J. Chen W. Li Z. Y. Wei S. Ma X. H. Lu N. H. . (2020). Discussion on the development of pineapple industry and its export strategy in zhanjiang. Qual. Saf. Inspection Testing. 30, 107–110.

[B39] Zhao P. Ge X. Zi B. Ge Q. J. (2016). Planar linkage synthesis for mixed exact and approximated motion realization via kinematic mapping. J. Mech. Robotics. 8, 051004. doi: 10.1115/1.4032212

